# Biochemical Properties, Antioxidant Activity, and In Vitro Ruminal Fermentation of Four Medicinal Plant Species Grown in Northwestern Tunisia

**DOI:** 10.3390/molecules30224451

**Published:** 2025-11-18

**Authors:** Monia Bouzazi, Houcine Selmi, Fatma Tajini, Mourad Jridi, Selim Jallouli, Abid Ouerghui, Hichem Sebai

**Affiliations:** 1 Laboratory of Functional Physiology and Valorization of Bio-Resources (LR23ES08), Higher Institute of Biotechnology of Béja, University of Jendouba, P.O. Box 382, Béja 9000, Tunisia; monia_iamm@yahoo.fr (M.B.); jridimourad@gmail.com (M.J.); ouerghiabid@gmail.com (A.O.); sebaihichem@yahoo.fr (H.S.); 2 Laboratory of Sylvo-Pastoral Resources, Sylvo-Pastoral Institute of Tabarka, University of Jendouba, P.O. Box 345, Tabarka 8110, Tunisia; houcine_selmi@live.fr; 3 Laboratory of Bioactive Substances, Center of Biotechnology of Borj Cedria, P.O. Box 901, Hammam-Lif 2050, Tunisia; selimjallouli80@gmail.com

**Keywords:** medicinal plant extracts, biochemical composition, phenolic compounds, fermentation, volatile fatty acids

## Abstract

This study assesses the phytochemicals and bioactivity of four plant species: *Polypodium vulgare*, *Chamaemelum nobile*, *Ocimum forsskaolii* Benth, and *Lavandula stoechas*. Plant chemical composition was determined. Antioxidant activity was assessed using the DPPH assay, and HPLC identified key phenolic compounds. In vitro ruminal fermentation trials evaluated gas production, metabolizable energy (ME), organic matter digestibility (OMd), and the production of volatile fatty acids (VFAs), which were subsequently identified using GC–MS. Significant variations (*p* ≤ 0.05) were observed among species. *C. nobile* and *O. forsskaolii* exhibited the highest total nitrogen contents (3.39 ± 0.42% and 3.20 ± 0.69%, respectively). All species contained high levels of insoluble dietary fiber, with *L. stoechas* showing the highest neutral detergent fiber (62.39%). *C. nobile* also recorded the highest polyphenol and flavonoid levels (73.88 ± 0.79 mg GAE/g DW and 27.85 ± 0.54 mg QE/g DW, respectively), along with strong antioxidant activity (IC_50_ = 0.38 mg/mL). HPLC identified catechol in *P. vulgare*, ferulic acid in *C. nobile*, chlorogenic acid in *O. forsskaolii*, and apigenin in *L. stoechas* as major compounds. For in vitro fermentation, the highest gas production was recorded at D20, accompanied by increases in ME, OMd, and VFA production. GC-MS analysis revealed that the distribution of total VFAs among acetate, propionate, butyrate, and branched-chain fatty acids varied in a clear dose-dependent manner, closely associated with the acetate-to-propionate (A/P) ratio, which in turn influenced CH_4_ production. These findings highlight the potential of plant extracts rich in dietary fiber and bioactive compounds to offer a sustainable alternative to conventional feed additives, enhancing ruminal fermentation, energy efficiency, and livestock performance.

## 1. Introduction

The ethnobotanical use of medicinal plant species is an ancient practice worldwide, particularly in economically disadvantaged communities and remote regions. In recent decades, research on natural compounds has significantly accelerated, focusing on the extraction, analysis, and integration of these phytocompounds for potential applications in the cosmetic, pharmaceutical, and agri-food industries. However, despite their potential as natural antioxidants, their use in animal nutrition remains limited, which could improve the quality of animal products and contribute to human health. Beyond their nutritional and health benefits, certain medicinal plants may also play a role in environmental sustainability. Livestock is responsible for about 12% of global greenhouse gas (GHG) emissions, primarily due to methane production during ruminal fermentation [1]. Addressing this issue has become a key focus in climate change mitigation strategies, with increasing attention being paid to managing ruminal methane emissions [2]. In small ruminants, effective microbial digestion is essential to meet the nutritional needs of high-production animals. However, this digestion remains partially incomplete, leading to nitrogen losses (mainly in the form of urine and milk) and energy losses in the form of methane, with significant environmental and economic consequences. These losses highlight the urgency of optimizing digestive processes to improve animal performance while reducing emissions [3]. However, existing strategies such as the use of chemical additives and dietary modifications have shown limited results in terms of their long-term effectiveness and environmental impact [4]. In this context, the integration of medicinal plants into animal feed is emerging as a promising alternative, addressing multiple challenges including food security, GHG reduction, and the adaptation of livestock systems to climate change [5]. Furthermore, in agreement with Iommelli et al. [6], several studies have demonstrated that the inclusion of medicinal plants such as garlic, oregano, and rosemary in ruminant diets can significantly improve not only animal performance but also the nutritional quality of animal-derived products. Research has shown that these plants can regulate ruminal function, lower methane emissions, modulate ruminal fermentation, and improve digestive efficiency in ruminants [7].

Comprising 2103 species across 115 families and 742 genera, the Tunisian flora represents an underexploited resource with the potential to offer innovative applications. This potential is emphasized by this flora’s traditional ethnobotanical uses, particularly in veterinary care and herbal medicine, highlighting its broad utility [8]. The growing use of these plants can be attributed to high levels of bioactive compounds with antimicrobial, antiparasitic, and anti-inflammatory properties, which can play a vital role in improving ruminant health and boosting livestock performance.

Among these plants, *P. vulgare* (Polypodiaceae) is known for its anti-inflammatory properties and has been traditionally used in human medicine to treat respiratory ailments, digestive disorders, and skin diseases [9]. *C. nobile* (Asteraceae) is valued for its digestive and calming effects and has been widely consumed as an infusion to relieve gastrointestinal discomfort, anxiety, and skin inflammations [10]. *O. forsskaolii* Benth (Lamiaceae) is recognized for its antimicrobial and analgesic properties and has been employed in folk medicine to combat infections, support digestion, and alleviate pain [11]. *L. stoechas* (Lamiaceae) is appreciated for its antiseptic and expectorant effects and has been extensively used in the treatment of respiratory conditions, nervous disorders, and skin infections, while also being incorporated into cosmetic applications [12]. Given their well-documented medicinal properties in humans, these plants have also attracted interest for their potential applications in animal health and nutrition. In this context, the present study aims to characterize these four medicinal species (*P. vulgare*, *C. nobile*, *O. forsskaolii*, *and L. stoechas)* by analyzing their biochemical properties and assessing their digestibility through kinetic indicators of gas production during in vitro ruminal fermentation. We hypothesize that these aqueous plant extracts will enhance ruminal fermentation efficiency by improving fiber degradability and fermentation biomarkers, thereby contributing to more efficient ruminant nutrition and offering a natural and sustainable alternative to conventional livestock feeding practices.

## 2. Results

### 2.1. Chemical Composition of the Studied Plant Species

The results in Table 1 show that all parameters varied significantly (*p* ≤ 0.05) between the plant species. Notably, *L. stoechas* exhibited the highest dry matter and ash contents relative to the other species. In contrast, *C. nobile* and *O. forsskaolii* recorded the highest total nitrogen levels, and consequently, the highest crude protein contents.

Furthermore, our analysis of insoluble dietary fibers assessed the levels of neutral detergent fiber (NDF), acid detergent fiber (ADF), acid detergent lignin (ADL), hemicellulose (HC), and crude cellulose (CC), and the results indicated that the plants were relatively rich in dietary fiber. Specifically, insoluble fiber levels across all plants ranged as follows: NDF (41.04–62.39% DM), ADF (23.94–40.22%DM), ADL (5.65–14.58% DM), HC (33.65–56.74% DM), and CC (12.55–34.5% DM). Except for its ADL fraction, *L. stoechas* has the highest levels of all insoluble fiber parameters; NDF and CC were the dominant fractions, with values of 62.39 ± 0.71 and 34.5 ± 0.97% DM, respectively.

### 2.2. Extraction Yield, Phenolic Compound Content, and DPPH Activity

As shown in Table 2, all parameters varied significantly among the species (*p* ≤ 0.05). The highest yields were recorded for *C. nobile* (16.22 ± 0.41), *O. forsskaolii* (17.04 ± 0.85%), and *L stoechas* (16.44 ± 0.62), while *P. vulgare* exhibited the lowest (12.42 ± 0.37%). Among the studied plants, *C. nobile* and *L. stoechas* exhibited the highest polyphenol levels (73.88 ± 0.79 and 72.69 ± 0.05 mg GAE/g DW, respectively). Conversely, *P. vulgare* showed the lowest polyphenol content (30.45 ± 0.26 mg GAE/g DW). Similarly, flavonoid levels varied significantly, with *C. nobile* showing the highest content (27.85 ± 0.54 mg QE/g DW), followed by *L. stoechas* and *O. forsskaolii* (20.11 ± 0.12 and 19.25 ± 0.20 mg QE/g DW, respectively). In contrast, *P. vulgare* presented the lowest flavonoid level (9.03 ± 0.24 mg QE/g DW).

An antioxidant test of plant extracts showed a significant difference (*p* ≤ 0.01) between the species, with the strongest antioxidant activities recorded for *O. forsskaolii* (IC_50_ = 0.36 mg/mL), *C. nobile* (IC_50_ = 0.38 mg/mL), and *L. stoechas* (IC_50_ = 0.40 mg/mL) (Table 2). In contrast, *P. vulgare* showed a weaker effect with an IC_50_ value of 1.79 mg/mL.

### 2.3. Compounds Profile Identified in the Aqueous Extract of P. vulgare, C.nobile, O. forsskaolii and L. stoechas

**Figure 1:**
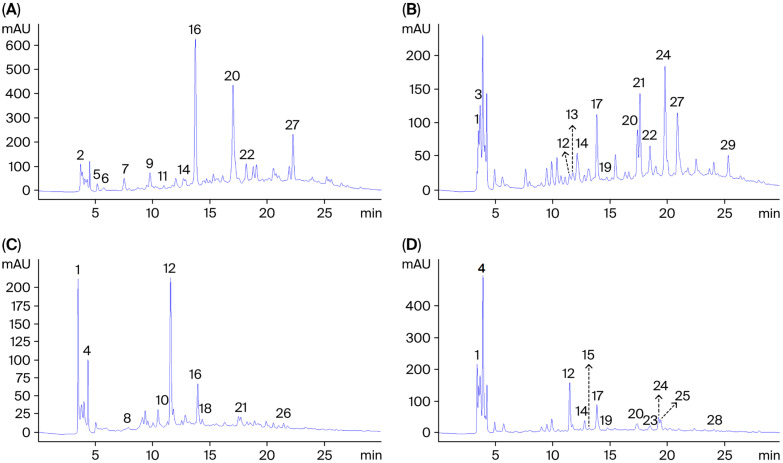
HPLC chromatogram of aqueous extracts from *P. vulgare* (**A**), *C. nobile* (**B**), *O. forsskaolii* (**C**) and *L. stoechas* (**D**). Peak numbers correspond to compounds in Table 3.

The HPLC analysis of the aqueous extracts from the studied plant species (Figure 1) revealed 11 compounds in *P. vulgare*, 13 in *C. nobile*, 9 in *O. forsskaolii*, and 12 in *L. stoechas*, classified into different phenolic acids and flavonoids (Table 3). Furthermore, in *P. vulgare*, catechol and ellagic acid were identified as the major compounds, accounting for 22.12% and 20.59%, respectively. In *C. nobile*, ferulic acid (10.42%) and apigenin-7-*O*-glucoside (9.39%) were the predominant compounds. Similarly, chlorogenic acid (23.53%) and ascorbic acid (14.22%) were the major constituents in *O. forsskaolii*. For *L. stoechas*, apigenin (21.40%) and chlorogenic acid (10.30%) were the dominant compounds.

### 2.4. Effects of Plant Extracts on the In Vitro Kinetics of Gas Production

**Figure 2:**
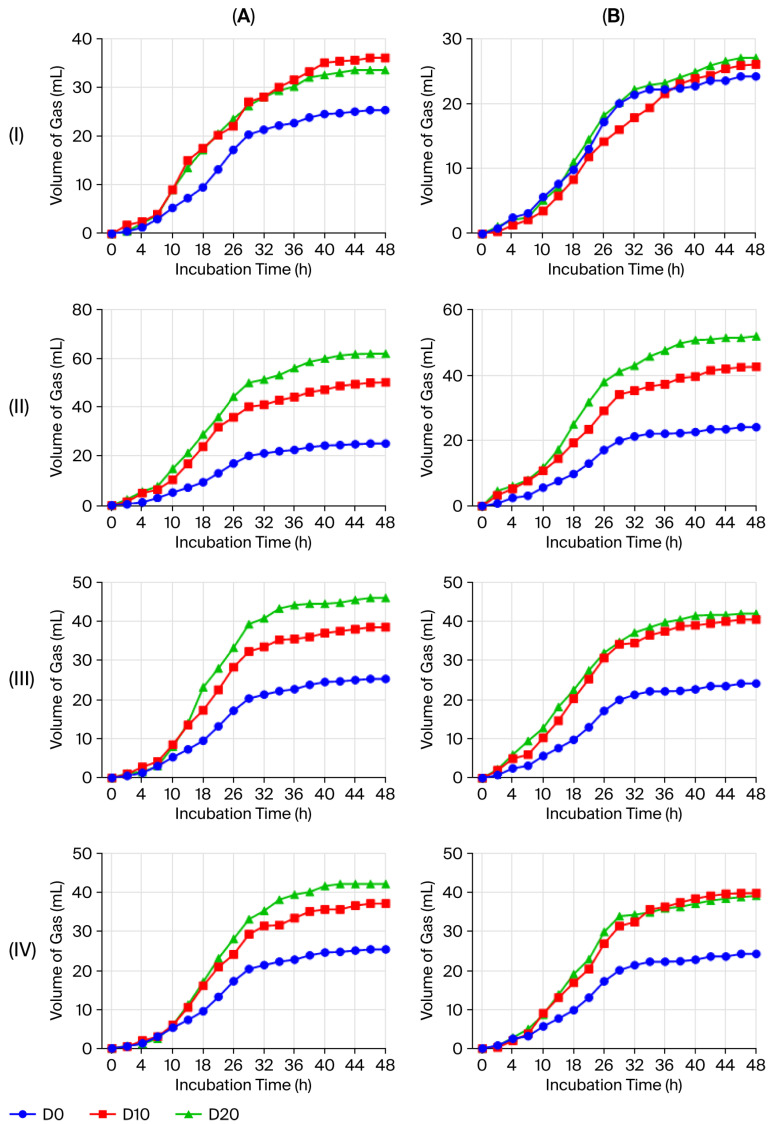
Kinetic curves for gas production induced by different extract doses of *P. vulgare* (**I**), *C. nobile* (**II**), *O. forsskaolii* (**III**), and *L. stoechas* (**IV**) in sheep (**A**) and goats (**B**). Values represent the means of three replicates performed on three animals for each species (sheep and goats).

The kinetic factors of in vitro fermentation are presented in Figure 2 and Table 4. For all substrates, the gas production curve follows three distinct phases: the lag phase (a), during which gas production remains minimal as rumen microbes adapt to the substrate; the exponential growth phase (b), characterized by rapid fermentation of the fermentable substrate fraction, leading to a consistent increase in gas production; and the plateau phase (c), where gas production slows and stabilizes, reflecting the rate of substrate degradation during this phase. All these parameters are crucial for assessing substrate digestibility and fermentation efficiency.

As shown in Table 4, gas production was mainly influenced by the degradation rate and characteristics of the carbohydrates in the feed, as well as variations in the plant species and extract doses.

The soluble fraction, “a”, showed significant variation between animal species. In sheep, the average values were generally lower, ranging from −5.38 ± 0.01 to −2.07 ± 0.01 mL at D20, whereas in goats, this parameter ranged from −3.38 ± 0.01 to −1.37 ± 0.01 mL at the same dose (D20). This parameter “a” corresponds to the gas volume extrapolated at *t* = 0. Negative values indicate the presence of a lag phase, reflecting the time required for microbial adaptation to the substrate.

Additionally, the fraction of potentially available fermentable substrate, “b”, varied significantly depending on plant species and doses. The highest values were observed with the *C. nobile* extract at D20 in sheep (112.70 ± 0.04 mL) and with *P. vulgare* at D10 in goats (395.30 ± 4.01 mL). Similarly, the rate of substrate degradation, “c”, varied between plant species and doses; the highest value was observed for *O. forsskaolii* extract with 0.029 h^−1^ at D20 in goats.

Furthermore, at D20, the highest gas production (Gp) values were recorded with *C. nobile*, reaching 62 mL in sheep and 52 mL in goats, followed by *O. forsskaolii* with 46 mL in sheep and 42 mL in goats. Interestingly, the greatest CH_4_ production at D20 was recorded with *O. forsskaolii* in sheep (24.83 ± 0.29 mL) and with *C. nobile* in goats (24.00 ± 2.00 mL). In contrast, the lowest CH_4_ values were observed with *P. vulgare*, while *L. stoechas* produced intermediate levels in both animal species.

### 2.5. In Vitro Impact of Plant Extracts on Ruminal Fermentation Parameters

As shown in Table 5, plant species, animal species, and extract dose had significant effects on metabolizable energy (ME) production, organic matter digestibility (OMd), and volatile fatty acids (VFAs) production. For all plant species, the D10 and D20 doses significantly improved these parameters compared with D0 in both animal species. Additionally, at D20, the *C. nobile* extract showed the highest values for all parameters. Sheep exhibited the greatest responses to the extract, with 7.85 ± 0.10 MJ/kg DM for ME, 52.49 ± 0.77% for OMd, and 0.90 ± 0.02 mmol for VFAs. In contrast, at the same dose, *P. vulgare* showed the lowest values across all parameters.

### 2.6. GC–MS Profile of Volatile Fatty Acids (VFAs)

After incubation, the rumen mixture from each treatment was subjected to GC-MS analysis, which identified ten compounds (Table 6). The major VFAs detected were acetic acid, propanoic acid, and n-butyric acid, while minor VFAs included isobutyric acid, 2-methylbutanoic acid, 3-methylbutanoic acid, and n-pentanoic acid. Additionally, other volatile compounds such as esters (isopropyl acetate, n-propyl acetate) and ketones (4-methyl-2-pentanone) were also observed. Only acetic acid was detected in *L. stoechas* at D20 (40.41%) in sheep, and at D20 in both *P. vulgare* (32.72%) and *L. stoechas* (38.53%) in goats.

On the other hand, in both animal species, the control predominantly exhibited acetate fermentation (acetic acid higher than propanoic acid), with an acetate-to-propionate (A/P) ratio > 1. Interestingly, in sheep at D10, all plant extracts promoted propionate fermentation (A/P ratio < 1); a similar effect was observed in goats, particularly with *C. nobile* and *L. stoechas*. By D20, however, fermentation shifted toward acetate (A/P ratio > 1) with all plant extracts in both animal species.

## 3. Discussion

This study aimed to characterize the phytochemical profiles of four medicinal plants—*P. vulgare*, *C. nobile*, *O. forsskaolii*, and *L. stoechas*—collected from wild populations in Northwestern Tunisia, and to evaluate their antioxidant activities and in vitro effects on ruminal fermentation in sheep and goats.

This investigation revealed higher mineral matter content in *L. stoechas* and significantly higher total nitrogen levels in *C. nobile* and *O. forsskaolii*. On the other hand, the various plant species were identified as potential sources of insoluble fibers, with *L. stoechas* showing the highest levels. These results were consistent with those reported for similar species, including other Asteraceae species and Mediterranean shrubs [13,14]. These fibers play a crucial role in digestive function, rumen health, and metabolic regulation, contributing to improved digestion, promoting the production of VFAs, which are key energy sources for ruminants [15], and influencing the quality of animal-derived products [16]. In livestock farming, ensuring appropriate fiber intake is essential, considering the animal type (ruminant or simple-stomach animals), its developmental or production stage, and its specific nutritional requirements [17].

Furthermore, total nitrogen (TN) plays a key role in ruminal fermentation by promoting microbial activity and protein synthesis, thereby enhancing fiber degradation and volatile fatty acids (VFAs) production. This is supported by Brinsi et al. [18], who reported a positive correlation between in vitro digestibility and TN content, highlighting the importance of nitrogen availability for efficient nutrient utilization.

In this study, we recorded elevated levels of phenolic compounds across the plant species, with *C. nobile* showing the highest values. Previous studies have demonstrated the variability in total phenolic content among these plant species. Indeed, *L. stoechas* has exhibited notably high phenolic content at 130.15 mg GAE/g of plant extract [19]. In contrast, *C. nobile* contained 1.72 mg GAE/g DW in green extracts [20]. Similarly, *O. forsskaolii* has been reported to have a phenolic content of 0.126 mg GAE/g DW [21]. The variability in phenolic compound levels observed in comparison with our results can be attributed to ecological and environmental factors, as well as differences in cultivation conditions, including the plant’s phenological stage and extraction methodologies. Therefore, the potent IC_50_ values observed in this study, particularly for *C. nobile*, *O. forsskaolii*, and *L. stoechas*, can be attributed to the high concentrations of phenolic compounds present in these plant species. Previous studies have reported a strong correlation between antioxidant activity and phenolic content, identifying these compounds as key indicators of antioxidant potential [22,23].

Regarding the diversity of natural phenolic compounds, the HPLC analysis performed in the present study enabled the identification of various phenolic acids and flavonoids. However, in contrast to our findings, previous studies reported that 3-*O*-caffeoylquinic acid [9], quercetin and rutin [24], caffeic acid and quercetin [11], and vanillic acid [25] are the most abundant compounds in *P. vulgare*, *C. nobile*, *O. forsskaolii*, and *L. stoechas*, respectively. These compounds have been identified as the primary contributors to the biological activities of these species.

The effect of the plant extract on ruminal fermentation was further assessed using the in vitro gas production technique. However, gas production was mainly influenced by the degradation rate and properties of the feed carbohydrates, along with differences in plant species and extract doses. The contribution of different carbohydrate fractions to feed digestibility and gas production was also reported by Calabrò et al. [26], who compared unfractionated forage samples, ethanol-insoluble residues (90% ethanol), and isolated neutral detergent fiber.

In both animal species, the highest gas production levels were recorded with *C. nobile*, followed by *O. forsskaolii* at the D20 dose, likely due to their higher total nitrogen content, elevated levels of phenolic compounds, and rich insoluble fiber content, which together enhance microbial activity and substrate fermentability in the rumen. These findings are consistent with those of Freitas et al. [27], who reported an increase in the soluble fraction parameter in response to plant extract supplementation in other ruminant species. Similarly, Gonzalez Ronquillo et al. [28] observed that higher plant extract doses led to a greater proportion of fermentable substrate available for ruminal fermentation. Previous studies have found that plant extracts can influence the rate of substrate degradation in some ruminants, depending on their bioactive compound content [27]. Particularly, flavonoid-rich extracts can stimulate specific microbial populations that are responsible for rapid substrate degradation and gas production in the rumen [29,30]. Messaoudi et al. [31] also confirmed that increasing the dose of plant extract improves fermentation speed by activating microbial processes in the rumen. These results reinforce the idea that bioactive plant compounds play a key role in optimizing ruminal fermentation processes and improving substrate digestibility in ruminants.

Methane (CH_4_) production in response to plant aqueous extracts was both dose- and species-dependent. *P. vulgare* slightly increased CH_4_ production in sheep, while *C. nobile* showed a biphasic effect, inhibiting CH_4_ at low doses but stimulating it at higher doses. At elevated doses, *O. forsskaolii* and *L. stoechas* mainly promoted CH_4_ production. These findings suggest that the impact of these extracts on rumen fermentation is strongly related to their bioactive composition. Our findings align with previous studies by Elghandour et al. [32] and Gao et al. [33], which demonstrated that aqueous extracts from plants such as *Azadirachta indica*, *Cnidoscolus angustidens*, and *Artemisia argyi* can modulate rumen fermentation and methanogenesis, underscoring their potential to mitigate methane emissions and promote livestock sustainability.

In our study, the higher values of metabolizable energy (ME), organic matter digestibility (OMd), and volatile fatty acids (VFAs) following the addition of plant extracts highlight their beneficial effects on ruminal fermentation. Moreover, the distribution of total VFAs among acetate, propionate, butyrate, and branched-chain fatty acids varied, with clear dose-dependent effects. In both animal species, low doses favored propionate fermentation, whereas higher doses shifted the balance toward acetate, highlighting the critical role of extract concentration [34,35]. These findings were closely linked to the acetate-to-propionate (A/P) ratio, where a lower A/P ratio reflects reduced methane production, while a higher A/P ratio corresponds to increased methane production. Overall, these findings demonstrate the beneficial role of plant extracts in modulating ruminal fermentation, confirming that their effects are influenced by plant species, dose and, to a lesser extent, by animal species, with potential implications for improving energy efficiency and mitigating methane emissions in ruminants [36,37,38].

Furthermore, the improvement in ruminal fermentation can be attributed to the functional interaction between insoluble fibers and bioactive compounds present in the studied plants, such as phenolic acids and flavonoids. However, fermentation efficiency is also influenced by the microbial composition of the rumen, which bioactive compounds can modulate by stimulating beneficial microorganisms. For instance, quercetin has been shown to enhance in vitro rumen fermentation by altering the microbial community structure, resulting in increased production of VFAs, particularly propionate—a metabolite known for its high energy efficiency in ruminants. This modulation also contributes to improved nitrogen utilization and a reduction in methane emissions [39]. Luteolin was also shown by Sinz [40] to reduce ruminal ammonia levels, contributing to better nitrogen efficiency.

On the other hand, Tánori-Lozano et al. [41] reported that ferulic acid enhances ruminal fermentation by promoting the degradation of structural and non-structural carbohydrates by stimulating the activity of cellulolytic and amylolytic bacteria, which increases the production of VFAs such as acetate, propionate, and valerate. It also exerts a mild antimethanogenic effect by partially inhibiting methanogenic archaea, redirecting metabolic hydrogen towards more energy-efficient fermentation pathways and reducing methane (CH_4_) emissions, an undesirable byproduct of ruminal fermentation. Additionally, ferulic acid reduces ruminal oxidative stress, supporting microbial growth and enzymatic activity, thus contributing to more stable and efficient fermentation. These combined effects improve carbohydrate utilization, energy efficiency, and selectively modulate rumen microbial populations.

In addition, ellagic acid and myricetin contribute to ruminal microbial balance by stimulating cellulolytic bacteria while inhibiting methanogenic microorganisms. This dual effect not only enhances fiber breakdown but also reduces methane emissions, thereby improving overall energy efficiency [4,42].

On the other hand, previous studies have highlighted the role of certain bioactive compounds, such as unsaturated fatty acids and tannins, in modulating ruminal fermentation and mitigating methane emissions. Unsaturated fatty acids, once in the rumen, undergo biohydrogenation, a process in which double bonds are saturated by hydrogen—thereby reducing hydrogen availability for methanogenesis. For instance, the inclusion of 5% rapeseed oil in forage-based rations for lactating dairy cows has been associated with up to a 23% reduction in methane production, without affecting the abundance of archaea and bacteria [43]. Tannins, natural polyphenols found in many plants, have also been shown to suppress methanogenesis by inhibiting cellulolytic microorganisms, reducing the acetate/propionate ratio, and decreasing ammonia concentrations through limited ruminal protein degradation. According to some evidence, tannins can reduce methane emissions by approximately 23% [44].

In summary, the observed effects of the studied plant extracts likely result from the synergy between various bioactive components, such as phenolic acids, flavonoids, insoluble fibers, and total nitrogen. This combination enhances cellulolytic bacterial activity, supports microbial protein synthesis, and modulates the ruminal microbial population by stimulating beneficial microbes. As a result, the degradation of complex fibers is improved, leading to increased VFA production and greater energy efficiency of ruminal fermentation, ultimately supporting better nutritional performance in ruminants. Importantly, these effects are dose-dependent, with lower extract levels contributing to a reduction in undesirable methane production.

Although our study was conducted in vitro, future investigations should evaluate the effects of varying forage-to-concentrate ratios and feed processing, as it is well established that such dietary factors significantly influence ruminal fermentation and methane production. An interesting avenue for future research would be to determine an optimal ratio for a mixture of the four plant extracts, in order to maximize their beneficial effects on ruminal fermentation and animal nutrition, while considering potential interactions between bioactive compounds. In particular, in vivo studies should consider the interaction between plant extracts and diet composition to better assess their combined impact on ruminal efficiency and methane mitigation. Furthermore, the potential presence of bioactive or toxic compounds in the plant should be investigated to ensure animal safety and to guide appropriate dosage recommendations.

## 4. Materials and Methods

### 4.1. Plant Material

Four spontaneous medicinal plants (*P. vulgare*, *C. nobile*, *O. forsskaolii*, and *L. stoechas*) were collected in March 2023 during their flowering stage from scrublands in the Aïn Draham area, located in northwestern Tunisia (36°46′ N, 8°41′ E; average elevation 574 m). The region is characterized by a humid climate, with an average annual rainfall of 1534 mm and mild winters, and an average annual temperature of 31.3 °C. The aerial parts of each plant were harvested and transported to the laboratory. Species identification was performed by Professor Marouani Ahmed from the Higher School of Agriculture of Kef (ESAK, El Kef, Tunisia), with voucher specimens (PAr1, CAr2, OAr3, and LAr4) stored for future reference within the Herbarium of the Laboratory of Functional Physiology and Bio-Resource Valorization at the Higher Institute of Biotechnology of Béja. Before conducting the analyses, the samples were air-dried at room temperature for twenty days.

### 4.2. Animal Material

Rumen fluid used for the in vitro incubation assays was collected from a total of six adult ruminants: three Barbarine rams (average body weight: 70 ± 0.6 kg) of the ovine species and three Alpine bucks (average body weight: 74 ± 1.4 kg) of the caprine species, all 26 months old, raised at the “Pure Nature” Agricultural Cooperative in northwestern Tunisia. The animal species were maintained on a standard diet composed of 70% concentrate and 30% oat hay for a period of 6 weeks, formulated to meet their maintenance nutritional requirements. The chemical composition of the diet was presented in Table 7.

The rumen fluid was collected immediately post-slaughter at the municipal slaughterhouse of Tabarka, in accordance with national legislation on animal welfare, specifically Law No. 2005-95 of October 18, 2005, relating to livestock and animal products [45]. It was then transferred into pre-warmed thermos flasks (maintained at approximately 40 °C using warm water) and promptly transported to the laboratory for use in the in vitro fermentation assays.

### 4.3. Phytochemical Analysis

#### 4.3.1. Dry Matter, Ash, Total Nitrogen and Dietary Fiber Determination

Dry matter content was quantified by drying 1 g of fresh sample at 105 °C until a constant weight was reached. Ash content was determined by incinerating 1 g of each sample at 550 °C in a muffle furnace for 4 h to ensure complete combustion. Total nitrogen (TN) content was determined using the Kjeldahl method. All analyses were performed in triplicate. The procedure comprised three main steps. First, samples underwent mineralization in an acidic medium using a Kjeldahl digester (BEHR LABORTECHNIK, Düsseldorf, Germany), with sulfuric acid and a copper sulfate (CuSO_4_) catalyst. Second, the resulting ammonium (NH_4_^+^) was converted into ammonia (NH_3_) through the addition of 10N sodium hydroxide, followed by distillation into a 4% boric acid solution containing mixed indicators (methyl red and bromothymol blue), using a semi-automatic nitrogen distillation unit (VELP Scientifica, Agrate Brianza, Italy). Lastly, the trapped ammonia was titrated with (0.98 mol·L^−1^) sulfuric acid until the end point was reached. Crude protein (CP) content was assessed using the standard conversion factor (TN × 6.25).

Dietary fiber was analyzed following the method outlined by Van Soest et al. [46] using a FIBRESTEST apparatus (RAYPA, Barcelona, Spain). Neutral detergent fiber (NDF) was assessed by dissolving cellular contents with sodium lauryl sulfate detergent, and pectic substances were solubilized with a chelating agent in a buffered solution. This fraction predominantly includes hemicelluloses, cellulose, and lignin. Acid detergent lignin (ADL) was measured by treating the acid detergent fiber (ADF) fraction, which consists of lignin and true cellulose, with sulfuric acid. Hemicellulose content was calculated by subtracting ADF from NDF, and cellulose content by subtracting ADL from ADF. Each sample was tested in triplicate.

#### 4.3.2. Preparation and Yield of Plant Extracts

The aerial parts of each dry plant were extracted in distilled water with a 1:10 (*w*/*v*) ratio for 48 h in the dark. After filtration through Whatman No. 4 filter paper (Sigma-Aldrich, St. Louis, MO, USA), the extracts were freeze-dried using a LABCONCO freeze-dryer (Kansas City, MO, USA). To ensure maximum yield, the extraction was repeated three times on the same plant residue. The extracts were stored in glass tubes at 4 °C, away from light. The yield (%) of dried extracts was calculated as DW_extr_/DW_samp_ × 100, where DW_extr_ is the weight of the extract after freeze-drying, and DW_samp_ is the dry weight of the sample.

#### 4.3.3. Total Polyphenol Determination

Polyphenol content was estimated using the Folin–Ciocalteu reagent, following the method described by Olsen and Sommer [47]. Absorbance was read at 760 nm with a UV–visible spectrophotometer (Jenway 7205, Staffordshire, UK), using a blank for the reference and gallic acid as the standard (y = 0.003C + 0.102; R^2^ = 0.971; concentrations were in the range of 0–800 µg/mL). The assay was performed in triplicate, and the total polyphenol content was represented as mg of gallic acid equivalent/g of dry weight (mg GAE/g DW) of the plant material.

#### 4.3.4. Total Flavonoid Determination

The flavonoid content was assessed using the method outlined by Tajini et al. [48]. Quercetin was used as a positive control (y = 0.372C − 0.011; R^2^ = 0.984; concentrations were in the range of 0–8000 µg/mL), and the absorbance was read at 510 nm with a UV–visible spectrophotometer (Jenway 7205, Staffordshire, UK), using a blank for calibration. The assay was performed in triplicate, and the flavonoid content was determined as mg of quercetin equivalent/g of dry weight (mg QE/g DW) of the plant material.

#### 4.3.5. Antioxidant Activity

The DPPH radical-scavenging assay was evaluated using a method adapted from Sarr et al. [49], and the activity was measured according to the following equation: % DPPH radical-scavenging effect = [(C_0_ − C_s_)/C_0_] × 100, where C_0_ and C_s_ are the concentration values of the DPPH solution before and after adding the extract sample, respectively. C_0_ and C_s_ were determined according to a calibration curve, and the absorbance at 517 nm was measured against a control, carried out with a methanolic DPPH solution. The extract concentration required to inhibit 50% of the DPPH radicals (IC_50_) was determined using a linear regression of the inhibition percentages. The IC_50_ assay was conducted in triplicate, and gallic acid (GA) was applied as a standard.

#### 4.3.6. HPLC Analysis of Plant Aqueous Extracts

The separation of phenolic compounds was performed using an Agilent 1100 series HPLC system (Santa Clara, CA, USA) equipped with an online degasser (G1322A), a quaternary pump (G1311A), a thermostatted autosampler (G1313A), a column oven (G1316A), and a diode array detector (G1315A). The system was operated using Chemstation software (version 10.1, Agilent) under Windows 2000. The analysis was carried out on a reverse-phase ODS C18 column (4 μm, 250 × 4.6 mm, Hypersil) maintained at ambient temperature. The mobile phase consisted of acetonitrile (solvent A) and sulfuric acid–acidified water (0.2% *v*/*v*) (solvent B), with a flow rate of 0.5 mL/min. The gradient program was as follows: 15% A/85% B (0–12 min), 40% A/60% B (12–14 min), 60% A/40% B (14–18 min), 80% A/20% B (18–20 min), 90% A/10% B (20–24 min), and finally 100% A (24–28 min). The injection volume was 20 μL, and detection was carried out at 280 nm. Compound identification was performed by comparing retention times and UV spectra with those of pure standards (Sigma, St. Louis, MO, USA).

### 4.4. In Vitro Evaluation of Gas Production

The fermentation of substrates in the rumen was evaluated using the in vitro gas production method, outlined by Jedidi et al. [50]. Briefly, the collected rumen fluid was blended, filtered using four layers of surgical gauze to eliminate any solid residues, and kept in an insulated container to maintain rumen-like conditions. The filtered fluid was subsequently transferred to an industrial mixer and an assay that was continuously exposed to CO_2_ to maintain anaerobic conditions. To prepare the fermentation medium, each 100 mL syringe was filled with a mixture of 10 mL of filtered rumen fluid, 20 mL of artificial saliva, and 0.3 g of ground oat hay as the base substrate. The artificial saliva, prepared according to McDougall (1948) [51], contained per liter: 9.8 g sodium bicarbonate (NaHCO_3_), 3.71 g disodium hydrogen phosphate (Na_2_HPO_4_), 0.57 g potassium chloride (KCl), 0.47 g sodium chloride (NaCl), 0.04 g calcium chloride (CaCl_2_), and 0.12 g magnesium sulfate (MgSO_4_), adjusted to pH 6.8–7.0 and used immediately or stored under CO_2_ to prevent oxidation. Additional syringes were prepared using the same components, with varying doses of plant aqueous extracts (AE) added separately (D0 = 0 µL, D10 = 10 µL, and D20 = 20 µL). Each assay was conducted in three independent runs (series), using rumen fluid from three animals per species (sheep and goats). The experimental design included four plant extracts (AE), three doses, and three technical replicates per treatment within each run, following a randomized arrangement.

After incubation at 39 °C in a water bath, the syringes were purged with CO_2_ to maintain anaerobic conditions. To ensure uniformity, the syringes were gently agitated for 2 h before measurements over a 48 h period. Gas volumes were recorded by holding the syringes horizontally without moving the piston for precise readings. Incubation ended when gas production stabilized. At the end of incubation, one sample from each treatment was stored at –20 °C for volatile fatty acid (VFA) analysis by GC–MS. For the remaining samples, 5 mL of NaOH (10 N) was injected into each syringe; the piston retracted due to CO_2_ absorption, and the resulting volume change was used to quantify methane (CH_4_) production.

Gas generation was modeled using the exponential equation Y = a + b(1 – exp −ct) [52], where “a” represents the gas production of the soluble part of the substrate (mL); “b” represents the gas production of the insoluble part of the substrate (mL); “a + b” represents the potential total gas production (mL), that is, the asymptote of the curve; “c” represents the gas production rate (in mL/hour), reflecting the fermentation speed; and “t” represents the culture time (in hours).

### 4.5. In Vitro Fermentation Parameters

Organic matter digestibility (OMd) was estimated according to the formula of Menke and Steingass [53]. Metabolizable energy (ME) content and total volatile fatty acids (VFA) production were calculated based on the method proposed by Makkar [54] using the following formulas:

OMd (%) = 14.88 + 0.889 Gp + 0.45 CP + 0.0651 MM(1)

ME (MJ/kg DM) = 2.20 + 0.136 Gp + 0.057 CP(2)

VFA (mmol/syringe) = 0.0239 Gp − 0.0601(3)

where Gp: volume of gas produced (mL/300 mg dry matter) after 24 h of incubation; CP: crude protein (g/100 g dry matter); MM: mineral matter (g/100 g dry matter).

### 4.6. GC–MS Analysis of Volatile Fatty Acids (VFAs)

The analysis was performed using a GC–MS system (Agilent) equipped with an HP-5MS capillary column (5% phenyl-methylpolysiloxane, 30 m × 0.25 mm × 0.25 µm). Manual injection was carried out in splitless mode; the injector was maintained at 250 °C, with a pressure of 9.7853 psi, a total flow of 19.2 mL·min^−1^, and a septum purge of 3 mL·min^−1^ (purge to split vent 15 mL·min^−1^ at 0.75 min). The oven temperature program was as follows: 50 °C (1 min), ramped at 3 °C·min^−1^ to 100 °C (1 min), then at 25 °C·min^−1^ to 300 °C (3 min), giving a total run time of 29.667 min (maximum temperature 325 °C). The MS transfer line was set at 280 °C, and the column flow was controlled in constant flow mode (initial 1.2 mL·min^−1^, post-run 0.67912 mL·min^−1^). Mass spectrometry was performed in scan mode (solvent delay 1.0 min) with a scan range of *m*/*z* 41–115, using electron impact (EI) ionization at 70 eV. Volatile fatty acids (VFAs) were identified by comparison with authentic standards.

### 4.7. Statistical Analysis

An analysis of variance (ANOVA) was used to detect significant differences among means, followed by Duncan’s multiple range test at a significance level of *p* ≤ 0.05. All statistical analyses were conducted using SAS software, version 8.2 (SAS Institute Inc., Cary, NC, USA) [55]. The assumptions of the ANOVA model, specifically data normality and homogeneity of variances, were considered during the analysis process.

## 5. Conclusions

The results showed that all plant species were relatively rich in dietary fiber, with *L. stoechas* exhibiting the highest levels. Regarding phenolic compounds, *C. nobile* and *L. stoechas* exhibited the highest polyphenol levels, with *C. nobile* also showing the highest flavonoid content compared with other species. Furthermore, strong antioxidant activities were recorded, and several phenolic acids and flavonoids were identified using HPLC analysis on all plant species.

In vitro substrate digestibility showed significant effects from plant species and extract doses, and the highest gas production values were observed at D20. Compared to control, all fermentation parameters (ME, OMd, and VFAs) showed significant improvements following the addition of plant extracts, demonstrating considerable potential for improving animal digestibility. These extracts modulated CH_4_ production in a dose- and species-dependent manner, with lower extract levels associated with reduced methane production. They also influenced ruminal fermentation by altering VFA distribution and adjusting the acetate-to-propionate ratio, which directly affects methanogenesis.

Overall, these plants represent promising solutions for sustainable livestock systems. By providing natural alternatives to conventional feed additives, they can enhance small ruminant health and productivity while supporting environmentally friendly animal nutrition. In vivo studies will be essential to confirm these findings, assess long-term effects on animal health, oxidative stress, and performance, and evaluate broader ecological benefits and their potential applications in livestock production.

## Figures and Tables

**Table 1:** Dry matter, ash, total nitrogen, and insoluble dietary fiber contents (% DM)) in the aerial parts of *P. vulgare*, *C. nobile*, *O. forsskaolii*, and *L. stoechas*.

Parameters	Content (%DM)			
	*P. vulgare*	*C. nobile*	*O. forsskaolii*	*L. stoechas*
DM	24.25 ± 0.04 ^b^	21.46 ± 0.03 ^c^	19.87 ± 0.17 ^c^	27.49 ± 0.12 ^a^
Ash	4.55 ± 0.01 ^ab^	3.99 ± 0.01 ^b^	3.67 ± 0.03 ^b^	6.11 ± 0.02 ^a^
TN	1.97 ± 0.08 ^b^	3.39 ± 0.42 ^a^	3.20 ± 0.69 ^a^	1.78 ± 0.14 ^b^
CP	12.30 ± 0.49 ^b^	21.16 ± 2.62 ^a^	20.02 ± 4.31 ^a^	11.15 ± 0.86 ^b^
NDF	48.23 ± 0.70 ^b^	41.04 ± 0.99 ^d^	43.48 ± 0.41 ^cd^	62.39 ± 0.71 ^a^
ADF	27.13 ± 0.11 ^b^	23.94 ± 0.24 ^c^	26.46 ± 1.01 ^b^	40.22 ± 1.12 ^a^
ADL	14.58 ± 0.06 ^a^	6.72 ± 0.05 ^bc^	7.92 ± 0.50 ^b^	5.65 ± 0.15 ^c^
HC	21.10 ± 0.59 ^a^	17.10 ± 0.75 ^b^	17.02 ± 0.60 ^b^	22.17 ± 1.84 ^a^
CC	12.55 ± 0.18 ^c^	17.22 ± 0.19 ^b^	18.54 ± 0.50 ^b^	34.50 ± 0.97 ^a^

Data are means ± SD of three replicates. For each parameter, in the same line, means followed by different letters are significantly different using Duncan’s multiple range test at *p* ≤ 0.05). DM: dry matter, TN: total nitrogen, CP: crude protein, NDF: neutral detergent fiber, ADF: acid detergent fiber; ADL: acid detergent lignin; HC: hemicellulose; CC: crude cellulose.

**Table 2:** Yield, phenolic compounds, and antioxidant activity of *P. vulgare*, *C.nobile*, *O. forsskaolii*, and *L. stoechas*.

Assay	*P. vulgare*	*C. nobile*	*O. forsskaolii*	*L. stoechas*
Yield (%)	12.42 ± 0.37 ^b^	16.22 ± 0.41 ^a^	17.04 ± 0.85 ^a^	16.44 ± 0.62 ^a^
Polyphenols (mg GAE/g DW)	30.45 ± 0.26 ^c^	73.88 ± 0.79 ^a^	60.70 ± 0.62 ^b^	72.69 ± 0.05 ^a^
Flavonoids (mg QE/g DW)	9.03 ± 0.24 ^c^	27.85 ± 0.54 ^a^	19.25 ± 0.20 ^b^	20.11 ± 0.12 ^b^
DPPH IC_50_ (mg/mL)	1.79 ± 0.08 ^a^	0.38 ± 0.002 ^b^	0.36 ± 0.006 ^b^	0.40 ± 0.003 ^b^

Data are means ± SD of three replicates. For each parameter, in the same line, means followed by different letters are significantly different using Duncan’s multiple range test at *p* ≤ 0.05),mg GAE/g DW: mg gallic acid equivalent/g of dry weight of the plant material, mg QE/g DW: mg of quercetin equivalent/g of dry weight of the plant material.

**Table 3:** HPLC identification of phenolic compounds (%) in aqueous extracts from *P. vulgare*, *C. nobile*, *O. forsskaolii*, and *L. stoechas*.

Peaks Numbers	Compounds ^a^	RT ^b^	*P. vulgare*	*C. nobile*	*O. forsskaolii*	*L. stoechas*
1	Ascorbic acid	3.69	-	5.81	14.22	9.44
2	Shikimic acid	3.71	2.52	-	-	-
3	Apigenine-7-*O*-glucoside	3.93	-	9.39	-	-
4	Apigenin	4.29	-	-	6.89	21.40
5	Gallic acid	5.18	0.88	-	-	-
6	5-*O*-caffeoylquinic acid	5.74	0.50	-	-	-
7	3-*O*-caffeoylquinic acid	7.51	2.29	-	-	-
8	Rosmarinic acid	7.83	-	-	1.06	-
9	Hyperoside	9.77	2.75	-	-	-
10	Kaempferol	10.44	-	-	3.76	-
11	3,5-Dicaffeoylquinic acid	10.98	0.41	-	-	-
12	Chlorogenic acid	11.50	-	0.99	23.53	10.30
13	Résorcinol	11.84	-	1.31	-	-
14	Catechin	12.70	1.37	3.92	-	2.56
15	Vanillic acid	13.29	-	-	-	0.66
16	Catechol	13.73	22.12	-	7.63	-
17	Epicatechin	13.94	-	5.80	-	7.63
18	Syringic acid	14.32	-	-	1.17	-
19	Caffeic acid	14.88	-	0.34	-	0.73
20	Ellagic acid	17.02	20.59	3.92	-	2.65
21	Luteolin	17.74	-	6.83	1.97	-
22	Sinapic acid	18.17	3.68	3.13	-	-
23	*p*-Coumaric acid	18.61	-	-	-	1.94
24	Ferulic acid	19.36	-	10.42	-	3.02
25	*m*-Coumaric acid	19.55	-	-	-	2.45
26	Quercetin	21.80	-	-	0.51	-
27	Myricetin	22.26	6.89	7.56	-	-
28	Cinnamic acid	24.22	-	-	-	0.41
29	Ferulic-1-*O*-glucoside acid	25.49	-	1.79	-	-

^a^ Identification was confirmed using authentic commercial standards, ^b^ retention time.

**Table 4:** Effect of aqueous extracts from *P. vulgare*, *C. nobile*, *O. forsskaolii*, and *L. stoechas*, in vitro on equation Y = a + b (1 – exp −ct) and the characteristic parameters of gas production kinetics in sheep and goats.

Animals	Plants	Doses (µL)	a (mL)	b (mL)	c (mL/h)	Gp at 24 h(mL)	Gp at 48 h(mL)	CH4 (mL)
Sheep	*P. vulgare*	D0	−1.36 ± 0.01 ^a^	61.82 ± 0.02 ^b^	0.012 ^b^	14.67 ± 0.29 ^b^	25.33 ± 0.29 ^b^	10.50 ± 0.50 ^b^
		D10	−1.19 ± 0.01 ^a^	76.10 ± 0.01 ^a^	0.015 ^b^	20.50 ± 0.50 ^a^	36.00 ± 0.00 ^a^	11.50 ± 1.32 ^ab^
		D20	−2.07 ± 0.01 ^b^	56.49 ± 0.04 ^b^	0.023 ^a^	21.17 ± 0.58 ^a^	33.50 ± 0.00 ^a^	13.83 ± 1.04 ^a^
	*C. nobile*	D0	−1.36 ± 0.01 ^a^	61.82 ± 0.02 ^c^	0.012 ^b^	14.67 ± 0.29 ^c^	25.33 ± 0.29 ^c^	10.50 ± 0.50 ^b^
		D10	−3.45 ± 0.01 ^b^	82.87 ± 0.01 ^b^	0.023 ^a^	35.17 ± 0.28 ^b^	50.33 ± 0.29 ^b^	8.17 ± 0.76 ^b^
		D20	−3.68 ± 0.01 ^b^	112.70 ± 0.04 ^a^	0.020 ^a^	40.50 ± 0.87 ^a^	62.00 ± 0.00 ^a^	22.00 ± 0.00 ^a^
	*O. forsskaolii*	D0	−1.36 ± 0.01 ^a^	61.82 ± 0.02 ^c^	0.012 ^b^	14.67 ± 0.29 ^c^	25.33 ± 0.29 ^c^	10.50 ± 0.50 ^b^
		D10	−3.18 ± 0.01 ^b^	69.00 ± 0.20 ^b^	0.022 ^a^	25.17 ± 0.29 ^b^	38.50 ± 0.00 ^b^	12.16 ± 1.26 ^b^
		D20	−5.38 ± 0.01 ^c^	82.81 ± 0.01 ^a^	0.023 ^a^	30.33 ± 0.28 ^a^	46.00 ± 0.00 ^a^	24.83 ± 0.29 ^a^
	*L. stoechas*	D0	−1.36 ± 0.01 ^a^	61.82 ± 0.02 ^c^	0.012 ^b^	14.67 ± 0.29 ^b^	25.33 ± 0.29 ^c^	10.50 ± 0.50 ^b^
		D10	−3.39 ± 0.01 ^b^	82.13 ± 0.31 ^b^	0.016 ^a^	22.33 ± 0.29 ^a^	37.00 ± 0.00 ^b^	16.00 ± 1.00 ^a^
		D20	−4.66 ± 0.05 ^c^	106.10 ± 0.01 ^a^	0.014 ^ab^	26.50 ± 0.50 ^a^	42.00 ± 0.00 ^a^	17.00 ± 0.01 ^a^
Goats	*P. vulgare*	D0	−1.42 ± 0.01 ^b^	50.38 ± 0.01 ^c^	0.016 ^a^	14.50 ± 0.50 ^a^	24.17 ± 0.29 ^a^	10.50 ± 0.50 ^a^
		D10	1.47 ± 0.01 ^a^	395.30 ± 4.01 ^a^	0.001 ^c^	13.17 ± 0.76 ^a^	26.00 ± 0.01 ^a^	9.33 ± 1.04 ^a^
		D20	−1.84 ± 0.01 ^b^	67.67 ± 0.01 ^b^	0.013 ^b^	16.17 ± 0.76 ^a^	27.00 ± 0.01 ^a^	10.0 ± 0.50 ^a^
	*C. nobile*	D0	−1.42 ± 0.01 ^b^	50.38 ± 0.01 ^b^	0.016 ^b^	14.50 ± 0.50 ^c^	24.17 ± 0.29 ^c^	10.50 ± 0.50 ^b^
		D10	−0.69 ± 0.01 ^a^	87.81 ± 0.03 ^a^	0.015 ^b^	25.50 ± 1.00 ^b^	42.66 ± 0.58 ^b^	12.33 ± 1.04 ^b^
		D20	−1. 90 ± 0.01 ^b^	93.12 ± 0.01 ^a^	0.020 ^a^	34.00 ± 0.50 ^a^	52.00 ± −0.00 ^a^	24.00 ± 2.00 ^a^
	*O. forsskaolii*	D0	−1.42 ± 0.01 ^a^	50.38 ± 0.01 ^b^	0.016 ^c^	14.50 ± 0.50 ^b^	24.17 ± 0.29 ^b^	10.50 ± 0.50 ^b^
		D10	−2.27 ± 0.01 ^b^	64.21 ± 0.01 ^a^	0.025 ^b^	28.33 ± 0.76 ^a^	40.50 ± 0.00 ^a^	13.33 ± 1.53 ^a^
		D20	−1.37 ± 0.01 ^a^	60.89 ± 0.41 ^a^	0.029 ^a^	29.83 ± 0.29 ^a^	42.00 ± 0.00 ^a^	14.33 ± 0.58 ^a^
	*L. stoechas*	D0	−1.42 ± 0.01 ^a^	50.38 ± 0.01 ^c^	0.016 ^b^	14.50 ± 0.50 ^c^	24.17 ± 0.29 ^c^	10.50 ± 0.50 ^b^
		D10	−2.52 ± 0.01 ^b^	177.9 ± 0.06 ^a^	0.007 ^c^	22.83 ± 0.29 ^a^	41.50 ± 0.00 ^a^	9.33 ± 0.50 ^b^
		D20	−3.38 ± 0.01 ^c^	64.45 ± 0.01 ^b^	0.025 ^a^	26.67 ± 0.58 ^a^	39.00 ± 0.00 ^a^	19.00 ± 1.50 ^a^
Plant effect			***	*	***	***	***	***
Animal effect			***	ns	ns	**	**	ns
Dose effect			***	**	***	***	***	***

^a^: gas production from the easily fermentable soluble fraction (mL); ^b^: gas production from the potentially fermentable insoluble fraction (mL); ^c^: gas production rate (mL/hour); Gp: total gas volume (mL) produced after 24 and 48 h of incubation. D0 = 0 µL; D10 = 10 µL; and D20 = 20 µL of plant aqueous extracts. All values are expressed as mean ± SD from three replicates performed on three animals for each species (sheep and goats); for each parameter and plant species, the means followed by different letters in the same column are significantly different at *p* ≤ 0.05. *** *p* < 0.001, ** *p* < 0.01, * *p* < 0.05, ns: not significant *(p* > 0.05).

**Table 5:** Effect of aqueous extracts from *P. vulgare*, *C. nobile*, *O. forsskaolii*, and *L. stoechas* on metabolizable energy (ME), organic matter digestibility (OMd), and volatile fatty acids (VFAs) production in sheep and goats.

Plants	Doses	ME (MJ/kg DM)		OMd (%)		VFA (mmol)	
		Sheep	Goats	Sheep	Goats	Sheep	Goats
*P. vulgare*	D0	4.33 ± 0.04 ^c^	4.31 ± 0.07 ^a^	29.53 ± 0.26 ^b^	29.38 ± 0.44 ^b^	0.29 ± 0.01 ^b^	0.28 ± 0.01 ^b^
	D10	5.13 ± 0.07 ^a^	4.13 ± 0.10 ^a^	34.71 ± 0.44 ^a^	28.19 ± 0.68 ^b^	0.43 ± 0.01 ^a^	0.25 ± 0.02 ^b^
	D20	5.22 ± 0.08 ^a^	4.54 ± 0.10 ^a^	35.30 ± 0.51 ^a^	30.86 ± 0.68 ^a^	0.45 ± 0.01 ^a^	0.33 ± 0.02 ^a^
*C. nobile*	D0	4.33 ± 0.04 ^c^	4.31 ± 0.07 ^c^	29.53 ± 0.26 ^c^	29.38 ± 0.44 ^c^	0.29 ± 0.01 ^c^	0.28 ± 0.01 ^c^
	D10	7.12 ± 0.04 ^b^	5.81 ± 0.14 ^b^	47.75 ± 0.26 ^b^	39.16 ± 0.89 ^b^	0.78 ± 0.01 ^b^	0.55 ± 0.02 ^b^
	D20	7.85 ± 0.12 ^a^	6.96 ± 0.07 ^a^	52.49 ± 0.77 ^a^	46.71 ± 0.44 ^a^	0.90 ± 0.02 ^a^	0.75 ± 0.01 ^a^
*O. forsskaolii*	D0	4.33 ± 0.04 ^c^	4.31 ± 0.07 ^b^	29.53 ± 0.26 ^c^	29.38 ± 0.44 ^b^	0.29 ± 0.01 ^c^	0.28 ± 0.01 ^b^
	D10	5.76 ± 0.04 ^b^	6.19 ± 0.10 ^a^	38.86 ± 0.25 ^b^	41.68 ± 0.68 ^a^	0.54 ± 0.01 ^b^	0.62 ± 0.02 ^a^
	D20	6.49 ± 0.01 ^a^	6.40 ± 0.03 ^a^	43.60 ± 0.02 ^a^	43.05 ± 0.22 ^a^	0.66 ± 0.01 ^a^	0.65 ± 0.01 ^a^
*L. stoechas*	D0	4.33 ± 0.04 ^c^	4.31 ± 0.07 ^b^	29.53 ± 0.26 ^c^	29.38 ± 0.44 ^c^	0.29 ± 0.01 ^c^	0.28 ± 0.01 ^c^
	D10	5.38 ± 0.04 ^b^	5.44 ± 0.04 ^b^	36.34 ± 0.26 ^b^	36.79 ± 0.26 ^b^	0.47 ± 0.01 ^b^	0.48 ± 0.01 ^b^
	D20	5.94 ± 0.07 ^a^	5.97 ± 0.08 ^a^	40.05 ± 0.44 ^a^	40.19 ± 0.51 ^a^	0.57 ± 0.01 ^a^	0.57 ± 0.01 ^a^
Plant effect		***		***		***	
Animal effect		*		*		*	
Dose effect		***		***		***	

Data are expressed as mean ± SD from three replicates performed on three animals for each species (sheep and goats); for each parameter and each plant species, the means followed by different letters in the same column are significantly different at *p* ≤ 0.05. ME: metabolizable energy; OMd: organic matter digestibility;VFA: volatile fatty acids. D_0_ = 0 µL; D_10_ = 10 µL; and D_20_ = 20 µL of plant aqueous extracts. *** *p* < 0.001, * *p* < 0.05.

**Table 6:** GC–MS profile of volatile fatty acids from in vitro ruminal fermentation.

Animals	Plants	Doses (µL)	Compounds (%)										
			Acetic Acid	Propanoic Acid	n-Butyric Acid	Isobutyric Acid	3-Methylbut- Anoic Acid	2-Methylbut- Anoic Acid	n-Pent- Anoic Acid	Isopropyl- Acetate	n-Propyl- Acetate	4-Methyl-2- Pentanone	A/P Ratio
Sheep	Control	D0	23.96	5.23	29.11	4.02	6.88	6.52	5.52	0.76	4.12	11.89	4.58
	*P. vulgare*	D10	8.85	17.71	32.44	3.78	6.24	6.21	4.94	1.00	4.93	2.97	0.50
		D20	21.61	13.59	39.95	3.48	7.28	5.96	—	1.29	1.75	—	1.59
	*C. nobile*	D10	6.01	19.00	32.55	—	6.42	6.00	4.92	1.02	4.85	—	0.32
		D20	22.67	16.96	35.03	3.51	6.7	5.52	3.83	1.44	1.22	—	1.34
	*O. forsskaolii*	D10	14.7	22.95	25.98	2.43	3.75	3.15	2.69	0.49	—	—	0.64
		D20	33.66	15.95	37.24	2.41	3.21	1.35	—	1.69	1.64	0.55	2.11
	*L. stoechas*	D10	26.2	—	31.59	3.36	6.02	5.62	4.92	0.84	3.77	10.88	—
		D20	40.41	—	—	—	—	—	—	—	—	—	—
Goats	Control	D0	17.07	13.67	29.15	8.40	4.45	4.74	4.45	2.85	14.32	0.90	1.25
	*P. vulgare*	D10	10.73	—	29.21	—	4.42	5.75	—	6.88	27.25	2.02	—
		D20	32.72	—	—	—	—	—	—	—	—	—	—
	*C. nobile*	D10	4.38	10.79	32.61	—	4.76	5.29	4.24	4.65	20.66	1.43	0.40
		D20	9.69	11.29	31.29	11.03	6.07	7.48	—	4.15	17.75	1.25	0.86
	*O. forsskaolii*	D10	18.63	5.07	28.16	5.35	3.98	4.13	—	3.67	19.92	1.34	3.67
		D20	35.00	12.49	22.47	6.44	4.03	4.12	3.50	2.59	11.58	0.84	2.48
	*L. stoechas*	D10	14.68	33.05	29.87	—	4.10	5.48	—	4.72	—	1.70	0.44
		D20	38.53	—	—	—	—	—	—	—	—	—	—

A/P ratio: acetate-to-propionate ratio.

**Table 7:** Chemical composition of the standard diet.

Components	Oat Hay	Concentrate
Dry matter (%)	85.00	92.00
Mineral matter (%)	7.28	8.09
Organic matter (%)	92.72	91.91
Crude protein (%)	2.53	23.41
NDF (%)	68.77	42.60
ADF (%)	36.43	8.80

## Data Availability

Data are contained within the article.
